# Are ultra-processed foods too tasty? Toward a metabolic framework for diet and obesity

**DOI:** 10.1371/journal.pmed.1005025

**Published:** 2026-04-03

**Authors:** David S. Ludwig

**Affiliations:** 1 New Balance Foundation Obesity Prevention Center, Division of Endocrinology, Boston Children’s Hospital, Boston, Massachusetts, United States of America; 2 Department of Pediatrics, Harvard Medical School, Boston, Massachusetts, United States of America; 3 Department of Nutrition, Harvard T.H. Chan School of Public Health, Boston, Massachusetts, United States of America; 4 Clinical Research, Steno Diabetes Center Copenhagen, Herlev, Denmark

## Abstract

In this Perspective, David Ludwig outlines why, in light of a recent lawsuit against “hyper-palatable” ultra-processed foods (UPF), a new framework centered on the metabolic effects of food is required to address the links between UPF and obesity-related chronic disease.

## Teaser

A recent lawsuit against “hyper-palatable” ultra-processed foods has amplified controversies over its effects on obesity-related chronic disease. Addressing this public health crisis requires a new framework, centered on the metabolic effects of food.

On 2nd December, 2025, the San Francisco City Attorney filed a lawsuit on behalf of the people of California, alleging that several large food companies knowingly marketed addictive, “hyper-palatable” ultra-processed foods (UPF). Modeled after legal actions against the tobacco industry, the lawsuit asserts that these companies colluded “with the intent of hacking human biological instincts and processes and driving increased consumption of their UPF” to boost sales and profits. The complaint seeks to bar deceptive marketing practices, mandate corrective actions, and obtain restitution and civil penalties for the harms and healthcare costs associated with UPF.

Lawsuits like this may play a role in the public health campaign against chronic disease, especially when targeting manipulative marketing practices. The First Amendment to the US constitution does not give food companies the right to advertise harmful products to children. Nonetheless, responsibility for the dysfunctional food supply extends beyond the food industry. An honest historical appraisal spanning science, government, and industry will be necessary for litigation—and, more importantly, for public health efforts—to succeed.

## Faulty science, misguided policy

Obesity has been historically viewed as an energy balance disorder, with calorie restriction the nearly universal approach to treatment. However, the long-term success of calorie-reduced diets is notoriously poor, due in part to physiological responses that oppose continued weight loss [1].

In the 1980s, coincident with the onset of the obesity epidemic, the field of nutrition shifted its focus to fat restriction. The Surgeon General’s Report on Nutrition and Health (1988) identified reduction of fat intake as the “primary priority for dietary change”, largely because this macronutrient “contributes more than twice as many calories as … protein or carbohydrate”. In contrast, concern about sugar was limited to children and their susceptibility to dental caries.

Informed by this perspective, the US government called upon the food industry, in *Healthy People 2000* (1990), to “Increase to at least 5,000 brand items the availability of processed food products that are reduced in fat and saturated fat”. Accordingly, the industry replaced fat with refined starches and sugars—ingredients considered at worst benign [2]. To make these products palatable, the industry relied not only on sugar but also on a range of additives to simulate, or compensate for, the properties of fat. For instance, commercial nonfat yogurts may have stabilizers, thickeners, fillers, flavorings, and sweeteners, whereas full-fat yogurt typically contains only cultured milk.

Many items emblematic of UPF today were formulated during the low-fat diet era. In addition to reduced fat content, these products have lower energy density and fewer calories per serving, characteristics thought to promote spontaneous weight loss. The evidence suggests they have the opposite effect [3] – but not because of excessive tastiness.

## Hyper-palatability—a tautology in the conceptualization of UPF

Consistent with the Nova classification system [4], the San Francisco lawsuit describes UPF as comprising “food substances of no or rare culinary use, or classes of additives whose function is to make the final product sellable and often hyper-palatable”—that is, capable of causing addictive responses through pleasure and reward pathways. Indeed, Nova proponents consider hyper-palatability central to the mechanisms linking diet to obesity-related disease [4,5]. However, this notion is circular.

Hyper-palatable foods are defined as having nutrient combinations linked to overconsumption [4]. Why do these foods drive energy intake? Implicitly, because they are excessively palatable. However, the empirical support for this assumption is weak according to an authoritative review by de Araujo and colleagues [6], who conclude that “palatability affects what one eats but does not reflect how much one eats”. Nova recognizes that UPF may act through subconscious mechanisms unrelated to taste or sensory perception [4]. Even so, the concept of hyper-palatability, untethered as it is from objective measures of palatability, is at best unhelpful and potentially misleading. If UPF are hyper-palatable because of their nutrient content, why wouldn’t home-prepared (by definition, not ultra-processed) foods with similar nutrient combinations produce the same addictive behaviors? If an industrial additive is required for a food to be hyper-palatable (just one triggers the UPF designation), what plausible mechanism could mediate such a global effect across the chemically and functionally diverse range of common additives?

## The need for a metabolic framework

Many whole or minimally processed carbohydrates digest slowly because their food matrix limits enzyme access to constituent starches and sugars. Processing disrupts this matrix and accelerates the rate of digestion, as illustrated by comparing wheat berries with white bread and whole fruits with sugar (their primary caloric component). In contrast, fats and proteins are inherently digested more slowly, with far less distinction in healthfulness between whole (e.g., olives, steak) and processed (e.g., olive oil, hamburger) forms. By disregarding the differential effects of processing on macronutrients, Nova can obscure critical post-ingestive mechanisms.

To explore these mechanisms, a blinded, cross-over feeding study was conducted comparing two meals matched for energy, macronutrients, and sweetness: one with rapidly digestible, high-glycemic index carbohydrate and one with slowly digestible, low-glycemic index carbohydrate [7]. After the high- versus low-glycemic index meal, glucose and insulin responses were greater during the early postprandial period (0–2 h), whereas by 4 h glucose was lower and hunger ratings were higher. At that time point, the high-glycemic index meal elicited greater nucleus accumbens activity (as determined by functional magnetic resonance imaging), a brain region implicated in craving, reward, and addiction. These findings illustrate how the metabolic effects of food (e.g., postprandial swings in blood glucose) can trigger not only homeostatic, but also hedonic, eating—independently of palatability. In fact, many commonly reported binge foods, such as bread, potato chips, popcorn, and sugary cereals, are relatively bland.

A central conundrum in nutrition is why people with obesity typically experience the hallmarks of starvation [1], including severe hunger and a decline in energy expenditure, soon after commencing a calorie-restricted diet when they still have sufficient adipose tissue energy stores to satisfy requirements for months. Numerous conceptual models focused on carbohydrate or hyperinsulinemia have attempted to answer this question, though none have received general acceptance [8–11]. Beyond obesity, processed carbohydrates have been linked to diabetes, cardiovascular disease, and other chronic conditions associated with insulin resistance, conceptualized by Reaven in the 1980s as *Syndrome X* [12]—the constellation of risk factors now termed metabolic syndrome. Research into this framework, which emphasizes metabolic responses to diet, should be prioritized.

## Implications for legislation and litigation

Effective public health action hinges on identifying precise intervention targets. In the context of UPF, definitional imprecision [13,14]—stemming from a flawed understanding of diet–disease relationships—may impede legislative and judicial efforts.

Nova is grounded in an ideology that endorses foods produced through traditional culinary or artisanal practices while classifying all other foods as UPFs, irrespective of their health effects. Under this system, food manufacturers could use any amount of sugar, refined flour, and salt, which are major drivers of chronic disease. However, they would be discouraged from using protein concentrates, various dietary fibers, flavors naturally found in food (e.g., vanillin), and other benign or healthful additives, imposing unnecessary burdens and costs. Moreover, it remains unclear how industry could pragmatically address the putative problem of hyper-palatability or how readily the public would embrace the implied solution – transition to a less tasty food supply.

Overall, UPF consumption is associated with multiple adverse outcomes. However, these associations may reflect the adverse metabolic effects of rapidly digestible carbohydrates rather than hyper-palatability per se. Governmental policies and legal settlements may be counterproductive if they require industry to reformulate packaged foods without targeting the causal drivers of disease.

## Conclusions

The obesity epidemic emerged despite (and perhaps because of) a nearly single-minded focus on calories and the most calorie-dense nutrient, fat. Lacking an operational definition pertaining to palatability, the notion of hyper-palatability—conceptualized as a central mechanism linking UPF and obesity—offers little beyond a recognition that some foods drive weight gain through various conscious and subconscious pathways. Moreover, there is little evidence to support the notion that the problem with modern industrial food is excessive tastiness.

For lawsuits to succeed, plaintiffs must take a clear-eyed view of the forces that shaped our dysfunctional food system, including faulty science and misguided governmental policies. The food industry, with its legions of lawyers, will be familiar with this history. For science to advance, a metabolic framework is needed, focused on how foods affect satiety relative to calories consumed. This framework promises to provide a strong foundation for preventive public health action.
